# Liver Regeneration: Analysis of the Main Relevant Signaling Molecules

**DOI:** 10.1155/2017/4256352

**Published:** 2017-08-30

**Authors:** Yachao Tao, Menglan Wang, Enqiang Chen, Hong Tang

**Affiliations:** Center of Infectious Diseases, West China Hospital, Sichuan University, Chengdu, China

## Abstract

Liver regeneration is a highly organized tissue regrowth process and is the most important reaction of the liver to injury. The overall process of liver regeneration includes three phases: priming stage, proliferative phase, and termination phase. The initial step aims to induce hepatocytes to be sensitive to growth factors with the aid of some cytokines, including TNF-*α* and IL-6. The proliferation phase promotes hepatocytes to re-enter G1 with the stimulation of growth factors. While during the termination stage, hepatocytes will discontinue to proliferate to maintain normal liver mass and function. Except for cytokine- and growth factor-mediated pathways involved in regulating liver regeneration, new substances and technologies emerge to influence the regenerative process. Here, we reviewed novel and important signaling molecules involved in the process of liver regeneration to provide a cue for further research.

## 1. Introduction

The liver, composed of parenchymal cells—hepatocytes—and nonparenchymal cells including endothelial cells, Kupffer cells, lymphocytes, and stellate cells, has a unique capacity to precisely regulate its growth and mass, which is particularly remarkable since hepatocytes are stable cells and rarely divide in the normal state, as they are quiescent in the G0 phase of the cell cycle [1]. However, their proliferative capacity is initiated in the case of liver tissue loss. There are two different regenerative models. Partial hepatectomy (two-thirds of the liver is removed) initiates a unique response, during which the remaining diploid hepatocytes enter into the cell cycle to compensate for the loss of liver tissue, taking about a week [2]. Another pattern of the regenerative model is established by insult, such as toxins and viral infection, during which all hepatocytes are hurt and oval cells are considered as potent stem cells to differentiate into hepatocytes and biliary cells. Both of the two patterns of liver regeneration will be involved in the review.

**Figure 1:**
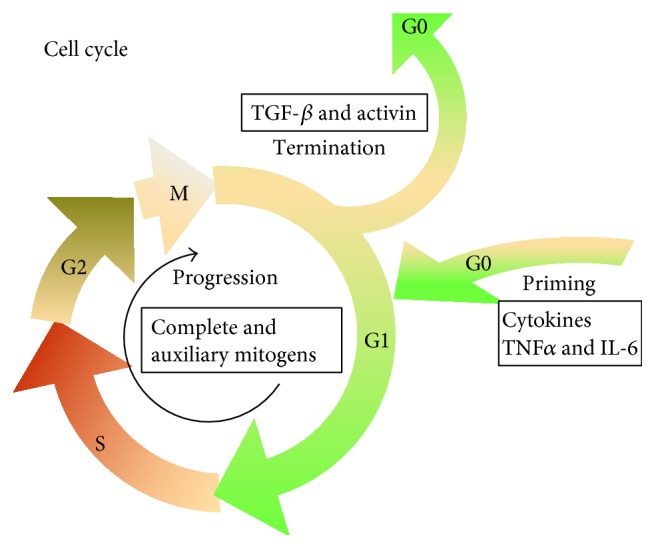
The outline of liver regeneration process.

Findings of past several decades have revealed that liver regeneration is a complex network regulated by various growth factors and cytokines expressed at the site of injury or migrated to the liver via the circulatory system. To sum it up, the regenerative process includes three critical steps [3]: firstly, quiescent hepatocytes convert from G0 to G1 of the cell cycle when faced with multiple stimulations (the priming phase); secondly, with the help of mitogens, hepatocytes progress beyond the restriction point to the G1 phase and then the mitosis (the proliferation phase); and then the last, cells terminate proliferation under the control of negative factors (the termination phase), such as transforming growth factor beta (TGF-*β*) and activin (Figure 1). In these three phases, various cytokines or growth factors exhibit a pivotal role through cell signaling pathways of multiple biological effects. Here, we endeavor to summarize some classical and novel signaling molecules participating in the process.

## 2. The Priming Phase: The Primary Molecules Tumor Necrosis Factor-*α* (TNF-*α*) and IL-6

Inflammation is a complex biological response and is characterized by recruitment, proliferation, and activation of a series of inflammatory cells and immune cells, and it aims to alleviate infections, eliminate damaged cells, and initiate tissue repair and regeneration [4]. Inflammation goes through the whole process of liver damage and promotes regeneration of the injured liver. Inflammation-induced regeneration primarily is triggered by cytokines and growth factors released from inflammatory cells. The most widely studied proinflammatory cytokines are TNF-*α* and IL-6.

**Figure 2:**
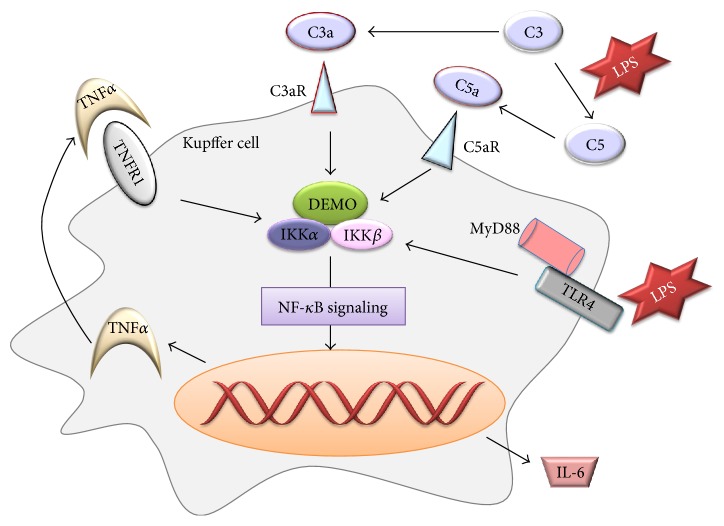
The production of TNF-*α* and IL-6 in Kupffer cell through NF-*κ*B signaling in the early phase of liver regeneration.

Kupffer cells are known to produce a group of cytokines and immunomodulating mediators that have stimulatory and inhibitory effects on hepatic injury. Hepatic macrophages are the main source of TNF-*α* and IL-6 through the NF-*κ*B signaling pathway triggered either by Gut-derived factor lipopolysaccharide (LPS)/Toll-like receptor4 (TLR4) signaling or by C3a and C5a, components of the complement system (Figure 2()). They prime hepatocytes to re-enter into the cell cycle in the first stage. Loss of either TNF-*α* or IL-6 could delay liver regeneration [5]. TLR4 recognizes its ligand LPS and then recruits and activates myeloid differentiation factor 88 (MyD88), triggering signal transduction downstream to promote the release of proinflammatory factors. In the view of C3 and C5, part of innate immune response which works in the process of liver injury to fight with multiple pathogens, they exert their effects on hepatocyte proliferation by activation of the bioactive peptides C3a and C5a with the stimulation of LPS [6] (Figure 2()). C3a not only mediates signals to the downstream C5a but also affects hepatocyte proliferation in a C5-independent fashion [7]. Mice deficient of either C3 or C5 showed impaired liver regeneration [8]. A complement inhibitor, CR2-CD59, targeting the site of complement activation and specially inhibiting the membrane attack complex (MAC), was used to study the complement-dependent balance between liver damage and regeneration and the results showed that CR2-CD59 not only has no effect on the production of C3a and C5a but enhances liver regeneration and remarkably improves the long-term survival, partly because of the increased level of hepatic TNF-*α* and IL-6 via STAT3 and Akt activation [6].

IL-6 is a pleiotropic cytokine and is secreted during inflammatory conditions upon LPS stimulation in a TNF-*α*-dependent/-independent manner (Figure 2). In response to liver injury, IL-6 mediates the acute-phase response and induces both cytoprotective and mitogenic functions. IL-6-induced signaling pathways are critical to the early onset as well as the progression and maintenance of the regenerative process [9]. Conventionally, IL-6 binds to the interleukin-6 receptor (IL-6R) and the IL-6/IL-6R complex initiates a coreceptor, glycoprotein (gp) 130, leading to JAk/STAT, MAPK, and PI3K/AKT activation [10]. STAT3 is able to upregulate the expression of suppressors of cytokine signaling (SOCS), an important negative regulator of cytokine signaling, leading to the downregulation of gp130 signals [10, 11]. Surprisingly, either IL-6 or IL-6R alone has no affinity to gp130 and only when the IL-6/IL-6R complex is formed, interaction with gp130 would occur.

Although gp130 is present in almost all cells, IL-6R is only expressed in limited cell types, for example, hepatocyte; thus, it seems that the effect of IL-6 is restricted to these cells. However, a soluble form of IL-6R (sIL-6R) was found and could still bind IL-6 to trigger intracellular signals, being called IL-6 trans-signaling [12]. SIL-6R is mostly generated by the proteolytic cleavage of membrane-bound receptor or by alternative splicing of the transmembrane domain coding exon [13]. The event of IL-6 trans-signaling not only could occur on cells short of IL-6R but also affects hepatocytes expressing IL-6R to prolong STAT3 phosphorylation and enhances the effect of IL-6 in liver regeneration [14, 15]. Blockade of IL-6 trans-signaling would deteriorate CCL4-induced liver damage [16]. It has been speculated that sIL-6R and sgp130, a soluble form of gp130, may constitute a buffer in the blood and once secreted, IL-6 will bind sIL-6R and then the complex IL-6/sIL-6R will bind sgp130 with a high affinity. Only when the concentration of IL-6 is very high, exceeding the level of sIL-6R, IL-6 could bind to membrane-bound IL-6R [17].

Of note, researches recently found that gp130, independent of the gp130 effector STAT3, initiates the activation of YAP and Notch, controlling the tissue growth and regeneration in intestinal epithelial cells upon mucosal injury [18]. Besides, YAP overexpression has been found in several solid tumors and elevated YAP levels contribute to tumor growth. YAP-mediated induction of Jag-1 was able to activate Notch signaling in HCC and mouse hepatocytes [19]. The role of this novel signaling in liver injury, repair, and regeneration remains to be charted.

## 3. The Proliferation Phase: Complete Mitogens and Auxiliary Mitogens

The proliferation phase, also called the second phase or progression phase, converts cells from G1 phase to mitosis. The molecules involved in the second phase were mainly separated into two groups, that is, complete mitogens and auxiliary mitogens [20] (Table 1). The former refers to these factors mitogenic in both primary-cultured hepatocytes and in animal experiments, including hepatocyte growth factor (HGF), transforming growth factor- (TGF-) *α*, epidermal growth factor (EGF), heparin-binding-EGF (HB-EGF), and their common receptor EGFR. They could activate secondary or delayed gene responses to stimulate DNA synthesis and cell proliferation. The latter, although they are not mitogenic in hepatocytes, may contribute to the regenerative process partially by magnifying or accelerating the effects of complete mitogens.

### 3.1. Complete Mitogens

**Figure 3:**
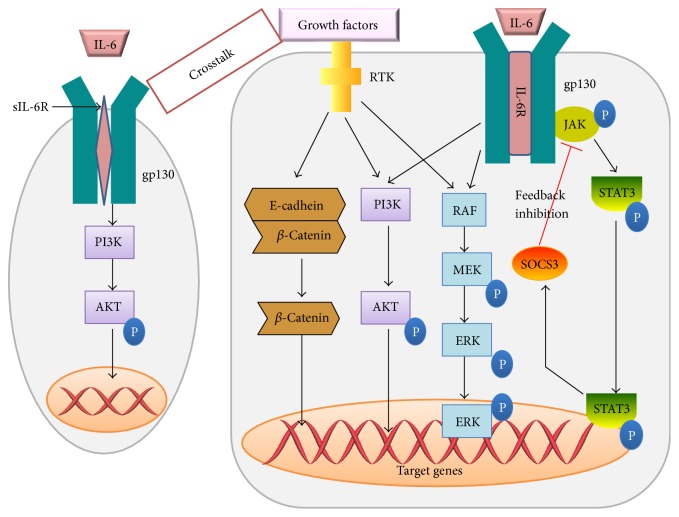
Growth factors, along with some cytokines, guide the progression of liver regeneration through expression of some cell cycle-related proteins mainly by PI3K/AKT, wnt-independent/*β*-catenin, Ras/MAPK, and JAK/STAT signaling pathways.

Complete mitogens exhibit direct hepatotrophic effects, which is defined that they could lead DNA synthesis in serum-free media in vitro and cause liver enlargement when injected in vivo. HGF and ligands of EGFR, including EGF, TGF-*α*, and HB-EGF, acting as the major complete mitogens for hepatocytes, could provoke hepatocyte proliferation mainly through the Ras-MAPK signaling and PI3K/AKT signaling pathway by binding to corresponding receptors, c-met and EGFR [21, 22] (Figure 3). Based on the research conducted by Huh et al. [23], mice knockout of the c-met gene showed hypersensitivity to Fas-mediated apoptosis and may retard the development of the liver after injury. The similar condition was also observed when EGFR was suppressed by silencing RNAs [24].

### 3.2. Auxiliary Mitogens

Auxiliary mitogens, such as bile acids (BAs) [25], norepinephrine (NE) [26], endothelial growth factor (VEGF) [27], insulin-like growth factor system (IGF system) [28], estrogen [29], and serotonin [30] (Table 1), although not mitogenic in cultured hepatocytes, may delay liver regeneration in their absence.

Blood platelets, not just functioning in the hematologic system, actually fulfill a wider role in health and diseases [42]. Platelets may be part of the innate immune system and also fight with infection, including bacteria, viruses, and microorganisms. Mediators provided by platelets not only recruit leukocytes to the site of vascular injury and inflammation but also aid in tissue repair and regeneration. Being recruited to the sinusoids after PHx and releasing molecules, such as HGF, VEGF, insulin-like growth factor-1 (IGF-1), and serotonin, platelets are described as a positive factor involved in liver regeneration [43]. Patients suffering from 70% PHx would improve the regenerative capacity of the liver if provided with plasma rich in platelets [44]. Conversely, administration of antiplatelet antibodies would depress liver regeneration [45]. However, it does not mean that administration of platelet concentrates and thrombopoietin receptor agonists could be widely used on clinical operations to support liver regeneration and alleviate outcomes of patients with liver failure or small-for-size syndrome owing to the severely undesirable side effects brought by the strategy, for example, venous or portal vein thrombosis, or even fatal transfusion-related acute lung injury [46]. Thus, more works are needed to ascertain its beneficial effects and to minimize potential side effects at the same time.

### 3.3. Wnt Proteins

Wnt ligands are secreted glycoproteins and are produced primarily by hepatic nonparenchymal cell compartment, especially Kupffer cells and endothelial cells [47]. They are beneficial and necessary for liver regeneration. Wnts may activate the chief downstream effector, *β*-catenin, and initiate the classic wnt/*β*-catenin signaling cascade and finally express target genes, such as *c-myc* and cyclinD1 [48]. Other than wnt proteins, *β*-catenin can also be stimulated through a non-wnt fashion, that is, wnt-independent signaling. *β*-Catenin forms the bridge between the cytoplasmic tail of E-cadherin and actin cytoskeleton, through which *β*-catenin may act as a mediator of tyrosine kinase signaling [49]. At the membrane, *β*-catenin could be phosphorylated at tyrosine residues 654 and 670 by different kinases including c-met, EGFR, and others [50], which induce the dissociation of *β*-catenin from E-cadherin, and subsequently, *β*-catenin translocates to the nucleus to control the expression of target genes (Figure 3()). Although both classic wnt/*β*-catenin signaling and wnt-independent signaling are advantageous to the regenerative process, the positive role of the former is more remarkable. When knocked out of LRP5/6, coreceptor of wnt proteins, mice showed impaired classic wnt/*β*-catenin signaling and retarded regenerative process after PHx despite that the non-wnt pathways remained intact [51].

### 3.4. Exosomes

Other than hormones, cytokines, and growth factors contributing to liver regeneration, exosomes are found to improve the regenerative process as well. Exosomes are membrane-enclosed nanovesicles possessing a variety of physiological properties and function as important vesicles involved in intercellular communication [52]. Exosomes are released by several types of cells and carry active signals to target cell within adjacent and remote areas. Recently, exosomes derived from hepatocytes were reported to improve liver regeneration owing to the production of intracellular sphingosine-1-phosphate (S1P) [53]. S1P is indispensable for hepatocyte exosome-induced proliferation. Only hepatocyte-derived exosomes, not other liver cells, contain neutral ceramidase and sphingosine kinase 2 (SK2) required for S1P synthesis. Exosomes fuse with and deliver synthetic machinery to target hepatocyte. And within the target hepatocyte, sphingosine-1-phosphate (S1P) is produced to promote cell proliferation. Besides, the number of hepatocyte-derived exosomes increased after liver injury. Similarly, MSC-derived exosomes exert hepatoprotective effects and relieve drug-induced liver injury through activation of proliferative and regenerative responses [54], underlining the tremendous potential of the exosome-based therapies for liver disease.

## 4. Termination of Liver Regeneration

When the normal liver mass/body mass ratio of 2.5% has been restored, liver regeneration would be terminated. However, mechanisms of controlling the hepatocyte apoptosis to correct an overshooting of regenerative response have not been well investigated. Thus far, the most well-known antiproliferative factors are transforming growth factor beta (TGF-*β*) and related TGF-*β* family members [55].

### 4.1. TGF-*β*-Mediated Pathways

TGF-*β*, especially TGF-*β*1, puts a brake on liver regeneration and works as an inducer of cell apoptosis in vitro and in vivo, being active in G1 phase of the cell cycle [56]. TGF-*β*1 exerts its function mainly through binding to its receptors type I receptor (T*β*RI) and type II receptor (T*β*RII) to encode related protein expression. Whereas, researchers recently have proved the lack of TGF-*β* gene upregulation in the termination stage and concluded that intact signaling by TGF-*β* may not be required for the termination phase of liver regeneration [57]. They also found that some genes were upregulated in the termination regulation and may have potential negative effect on the cell cycle and promotion of cell apoptosis, such as the zinc finger protein gene (ZNF490) and caspase recruitment domain-containing protein 11 (CARD11) gene. Therefore, more details are needed to verify and elucidate molecules that participated in the termination phase and the signaling they involved.

Apart from liver cells, TGF-*β*1 is also synthesized in extrahepatic tissues, including platelet and the spleen [58, 59]. The spleen, known as an immune organ, would secret TGF-*β*1 to end the liver regeneration. Splenectomy significantly increased the number of proliferating cells 48 h after PHx [60]. Recently, the spleen was proved to not only increase TGF-*β*1and its receptor T*β*RII but also downregulate HGF and its receptor c-met to exhibit growth inhibitory effects on cell proliferation, indicating that the spleen could remotely influence and regulate liver regeneration [59].

### 4.2. Other Relevant TGF-*β* Family Members

Other TGF-*β* family members, primarily activins and bone morphogenetic proteins (BMPs), were revealed to be implicated in numerous biological processes, including liver regeneration. Activin A, an activin subtype, is increased by 12 h in response to partial hepatectomy and considered as a negative regulator of liver regeneration and induces hepatocyte growth arrest and apoptosis in vitro and in vivo [61, 62]. Administration of the activin A antagonist follistatin enhanced DNA synthesis and prolonged hepatocyte proliferation [63]. Apart from the inhibitory effect on DNA synthesis, activin A also significantly affects the production of fibronectin, component of extracellular matrix (ECM) which is essential for liver regeneration [64]. With regard to BMP, unlike TGF-*β*1 and activin A, it is quite complicated. Major BMPs bind to their receptors mainly to phosphorylate Smad1/5/8 rather than Smad2/3 to exert repressive effects on liver regeneration [65]. However, different subunits of BMPs exhibit different or even reverse effects, for example, BMP7 that promotes hepatocyte proliferation, whereas BMP4 represses proliferation in the hepatoma cell line Huh7 [66]. One main possible reason may be that BMP7 and BMP4 act through pathways of opposite effects [67]. Thus, the accurate functions of other BMPs in the liver regeneration process remain to be undetermined.

## 5. The Future Perspectives in the Fields of Liver Regeneration

In spite of having been investigated for so many years, the actual mechanisms of liver regeneration are still obscure and far from practical application to solve clinical liver disease. However, the embarrassing situation is greatly improved by the appearance of new-fashioned technologies—cell transplantation therapy and liver bioengineering—aimed at alleviating the dilemma caused by insufficient liver regeneration or shortage of liver donors, and they are becoming hotspots in the research field. Cell transplantation, mainly referring to stem cells or progenitor cells, has been extensively studied on liver regeneration owing to the potential of differentiation into hepatocytes [68]. Studies have demonstrated that mesenchymal stem cells (MSC) [69], fetal progenitor cell [70], and embryonic stem cells [71] could improve liver injury to some extent. However, the source of transplanted cells and the livability and immune rejection after being transplanted may limit the application of cell therapy on clinical operations. Whereas, liver bioengineering, namely, three-dimensional matrix liver scaffolds, was first reported in 2010 and it includes two parts: decellularization and recellularization [72]. A decellularized liver scaffold (DLS) is characterized by retaining intact vasculature system and a fine web of matrix, providing necessary environment similar to a normal liver for cells to grow, proliferate, and differentiate [73]. After that, the DLS would be repopulated with functional human cells, mainly autologous liver progenitor cells. Furthermore, perfusion of the recellularized liver scaffold with positive molecules for cell regeneration or differentiation, for example, granulocyte colony stimulating factor (G-CSF), may facilitate liver regeneration [74]. Taken together, cell therapy, along with liver bioengineering, may be a new path for liver regeneration development.

## 6. Conclusions

Despite having been studied for so many years, the passion and energy for liver regeneration never fade out, since the demands for it is urgent from the past till now because of liver transplantation or liver failure or other end-stage liver diseases. It is a multifactor and multipath network, and the exact mechanisms are incompletely understood. Although the appearance of the new technologies opens our thoughts and horizons and, together with the previous results of researches, may drive us closer to clinical application, we still have a long way to go as we always operated studies on animals and the conclusions we deduced could not be applied to human directly because of species differences.

## Figures and Tables

**Table 1:** Common complete and auxiliary mitogens.

Factor	Origin	Target
*Complete mitogens*		
HGF	Mainly stellate cells	HGF directly regulates hepatocyte DNA synthesis and cell proliferation by blinding to its receptor c-met.
EGF	Brunner's gland in the duodenum	They provoke hepatocyte proliferation mainly through the Ras-MAPK signaling pathway by binding to their identical receptor and may compensate for each other to some degree in the process.
TGF-*α*	Hepatocytes	
*Auxiliary mitogens*		
Bile acids	Hepatocytes and cholangicytes	Appropriate concentration of BAs may promote liver regeneration mainly via farnesoid X receptor (FXR) signaling pathways to stimulate the expression of FoxM1b, a key regulator of cell cycle, to participate in cells proliferation [31].
NE	Nerve system	NE may amplify the effect of EGF and HGF by acting on the *α*1-adrenergic receptor associated with G*α*_h_, a G protein [32, 33], and besides, it could induced the expression of Smad7 to abolish activin A-induced growth inhibition of hepatocyte by activation of NF-*κ*B [34].
VEGF	Hepatocytes	VEGF family, particularly VEGF-A, is strongly upregulated in hepatocytes during the regenerative process and may facilitate proliferation of sinusoidal endothelial cells and hepatocytes 48 h following PHx [35].
Insulin	Pancreatic islets	Insulin could contribute to liver regeneration despite not being a primary mitogen and its proliferative effect mainly mediated through insulin receptors (IRs) that shift to nucleus to activate inositol 1,4,5,-trisphosphate- (InsP3-) dependent Ca^2+^ signaling pathways [36].
IGF-1	Liver	IGF-I works as a booster to liver regeneration by upregulation of HGF and downregulation of transforming growth factor beta 1(TGF-*β*1), a repressor of proliferation, and decreased level of IGF-I could impair the regenerative process [37].
Estrogen	Reproductive system	Estrogen has been shown to promote hepatocyte proliferation mainly through estrogen receptor alpha (ER*α*) [38]. Moreover, the estrogen level could be influenced by IL-6 and there may be crosstalk between estrogen signaling and IL-6 signaling pathways [39].
Serotonin (5-hydroxytryptamine, 5HT)	Enterochromaffin cells	Serotonin, via HT receptor 2 (HTR2), has been reported to contribute to liver regeneration [40]. And it was found that liver regeneration would be arrested when ketanserin was administrated to block 5-HT2, a subtype of 5-HT, approximately at the G1/S transition point [41].

## Abbreviations

TGF-*β*: Transforming growth factor beta
TNF-*α*: Tumor necrosis factor-*α*
TLR: Toll-like receptor
MyD88: Myeloid differentiation factor 88
AP-1: Activating protein1
LPS: Lipopolysaccharide
SOCS: Suppressors of cytokine signaling
HGF: Hepatocyte growth factor
TGF: Transforming growth factor
EGF: Epidermal growth factor
BAs: Bile acids
NE: Norepinephrine
VEGF: Endothelial growth factor
IGF: Insulin-like growth factor
ER: Estrogen receptors
ZNF490: Zinc finger protein gene
CARD11: Caspase recruitment domain-containing protein 11
ECM: Extracellular matrix
S1P: Sphingosine-1-phosphate
SK2: Sphingosine kinase 2
MSC: Mesenchymal stem cells
DLS: Decellularized liver scaffold
G-CSF: Granulocyte-colony stimulating factor.
